# Effectiveness of a walking programme to support adults with intellectual disabilities to increase physical activity: walk well cluster-randomised controlled trial

**DOI:** 10.1186/s12966-015-0290-5

**Published:** 2015-09-29

**Authors:** Craig A. Melville, Fiona Mitchell, Kirsten Stalker, Lynsay Matthews, Alex McConnachie, Heather M. Murray, Chris Melling, Nanette Mutrie

**Affiliations:** University of Glasgow, Institute of Health and Wellbeing, Academic Centre, Gartnavel Royal Hospital, 1055 Great Western Road, Glasgow, G12 0XH UK; Social Work and Social Policy, Lord Hope Building, University of Strathclyde, Glasgow, G4 OLT UK; MRC Social and Public Health Sciences Unit, University of Glasgow, 200 Renfield St, Glasgow, G2 3QB UK; Robertson Centre for Biostatistics, Institute of Health and Wellbeing, University of Glasgow, Boyd Orr Building, University Avenue Glasgow, Glasgow, G12 8QQ UK; Social Work Services, Glasgow City Council, 40 John Street, Glasgow, G1 1JL UK; University of Edinburgh, Physical Activity for Health Research Centre, St Leonard’s Land, Holyrood Road, Edinburgh, EH8 8AQ UK

## Abstract

**Background:**

Programs to change health behaviours have been identified as one way to reduce health inequalities experienced by disadvantaged groups. The objective of this study was to examine the effectiveness of a behaviour change programme to increase walking and reduce sedentary behaviour of adults with intellectual disabilities.

**Methods:**

We used a cluster randomised controlled design and recruited participants over 18 years old and not regularly involved in physical activity from intellectual disabilities community-based organisations. Assessments were carried out blind to allocation. Clusters of participants were randomly allocated to the Walk Well program or a 12-week waiting list control. Walk Well consisted of three face-to-face physical activity consultations incorporating behaviour change techniques, written resources for participants and carers, and an individualised, structured walking programme. The primary outcome measured with accelerometers was change in mean step count per day between baseline and 12 weeks. Secondary outcomes included percentage time per day sedentary and in moderate-vigorous physical activity (MVPA), body mass index (BMI), and subjective well being.

**Results:**

One hundred two participants in 50 clusters were randomised. 82 (80.4 %) participants completed the primary outcome. 66.7 % of participants lived in the most deprived quintile on the Scottish Index of Multiple Deprivation. At baseline, participants walked 4780 (standard deviation 2432) steps per day, spent 65.5 % (standard deviation 10.9) of time sedentary and 59 % percent had a body mass in the obesity range. After the walking programme, the difference between mean counts of the Walk Well and control group was 69.5 steps per day [95 % confidence interval (CI) -1054 to 1193.3]. There were no significant between group differences in percentage time sedentary 1.6 % (95 % CI −2.984 to 6.102), percentage time in MVPA 0.3 % (95 % CI −0.7 to 1.3), BMI −0.2 kg/m^2^ (95 % CI −0.8 to 0.4) or subjective well-being 0.3 (95 % CI −0.9 to 1.5).

**Conclusions:**

This is the first published trial of a walking program for adults with intellectual disabilities. Positively changing physical activity and sedentary behaviours may require more intensive programmes or upstream approaches to address the multiple social disadvantages experienced by adults with intellectual disabilities. Since participants spent the majority of their time sedentary, home-based programmes to reduce sitting time may be a viable health improvement approach.

**Trial registration:**

Current Controlled Trials ISRCTN50494254

**Electronic supplementary material:**

The online version of this article (doi:10.1186/s12966-015-0290-5) contains supplementary material, which is available to authorized users.

## Introduction

Finding ways to increase levels of physical activity and reduce sedentary time is an international public health priority [1] to reduce the global burden of non-communicable disease [2]. Behaviour change programs can have differential effects on the physical activity levels of socially advantaged and disadvantaged groups [3], unintentionally creating intervention generated inequalities [4]. One way to address this is for targeted trials to test the effectiveness of physical activity programmes in disadvantaged populations.

It is estimated that around 2 % of adults have intellectual disabilities [5], defined as significant limitations both in intellectual functioning and adaptive behaviour with onset before the age of 18 years [6]. Adults with intellectual disabilities experience multiple social disadvantage [7] and significant health inequalities [8]. Rates of obesity around 50 % [9] and an increased prevalence of diabetes experienced by adults with intellectual disabilities are in part explained by findings that around 5 % meet public health recommendations for levels of physical activity [10] and adults with intellectual disabilities spend around 85 % of their time sedentary [11]. This evidence on the poorer health and health behaviours of adults with intellectual disabilities means increasing levels of physical activity and reducing time spent sedentary is seen as a priority for reducing health inequalities [12].

Social, financial, transport and other barriers to accessing gyms [13, 14] mean that only a small proportion of adults with intellectual disabilities, who live in our communities, are able to regularly participate in gym-based programmes [15, 16]. No studies on non gym-based programmes have reported sedentary time as an outcome and the evidence is limited by small samples, uncontrolled designs and recruitment of biased samples [17]. Walking can be incorporated into everyday life [18], is a cheap and accessible form of physical activity and is the most common type of physical activity that adults with intellectual disabilities take part in [10, 11]. Therefore, programmes to increase walking may have fewer barriers to participation for adults with intellectual disabilities. Primary care and community based studies suggest that walking programmes can lead to significant health improvement [19]. However, no studies have examined the effectiveness of walking programmes in adults with intellectual disabilities.

To address this gap in the evidence base the overall aim of this study was to examine the effectiveness of a behaviour change programme to support adults with intellectual disabilities to walk more, to increase levels of physical activity and to reduce time spent sedentary.

## Methods

The trial used a two group, cluster-randomised controlled design in a sample of adults with intellectual disabilities. To examine the effectiveness of the 12-week Walk Well programme, data on the primary (mean steps/day) and secondary (moderate vigorous-vigorous physical activity, overall physical activity, sedentary behaviours, body mass index and wellbeing) outcomes were collected from participants allocated to the intervention group at baseline, upon completion of Walk Well at 12 weeks and after the end of the intervention (24-weeks) to examine maintenance effects. Participants allocated to the waiting list control group were invited to take part in Walk Well at the end of the 12-week waiting list period and post-intervention data collected to provide further information on the effects and acceptability of Walk Well. The trial was registered prior to data collection (http://www.isrctn.com/ISRCTN50494254) and the study protocol is described in full elsewhere [20]. The trial is reported according to CONSORT guidelines for reporting cluster randomised designs as outlined in Additional file 1.

## Ethical approval

Full ethical approval has been granted for the study by the Scotland A Research Ethics Committee (Reference 13/SS/229). In keeping with the Adults with Incapacity (Scotland) Act 2000, a participant with capacity provided their own written, informed consent and otherwise written consent to participation was provided by the nearest relative, or welfare guardian. The study sponsor was not involved in study design, collection and analysis of data, writing the report or the decision to submit the manuscript for publication.

## Study participants

Recruitment of participants took place between March 2013 and February 2014 and finished when the target sample size was reached. A multi-point strategy recruited participants from day centres for adults with intellectual disabilities, community provider organisations that employing paid carers and specialist intellectual disabilities health and social care services. Many adults with intellectual disabilities have frequent contact with other adults with intellectual disabilities such that there was felt to be a risk of participants in the intervention and control groups discussing the Walk Well programme, sharing resources or being influenced to change behaviours through direct contact. To minimise contamination of control group outcomes a cluster randomised design was used with participants randomised as part of a cluster if they attended the same day centre, lived in shared tenancies, or lived in different houses but were supported by the same paid carers.

Participants were eligible if they were over 18 years of age with any level of intellectual disabilities and excluded if they had severe challenging behaviour, required constant one-to-one support from carers or had significant mobility problems. Informed consent to participation was provided for all participants before data collection started.

## Randomisation

Clusters were the unit of randomisation. Baseline data for all participants in the cluster was complete before randomisation of the cluster. The researcher telephoned an interactive voice response system hosted by the Clinical Trial Unit to register the cluster in the study. Randomisation was stratified by the number of participants in the cluster (1, 2–3, > 4), to avoid an excessive imbalance between study groups. Within each stratum, the randomisation sequence was computer generated using the method of randomised permuted blocks, with a block length of 4 (2 intervention and 2 control). Allocations were revealed by telephone to the interactive voice response system, after baseline assessments had been made. The allocation sequence was known only to those managing the interactive voice response system, thereby concealing the next allocation in the sequence from researchers and participants. An automated email stating the allocation of the cluster (intervention or waiting list control) was sent to a member of the research team not involved in data collection (CAM) and the walking advisor notified.

## Sample size calculation

Prior to the study there was no step-count data from walking intervention studies for adults with intellectual disabilities. An average count of 6508 steps per day (standard deviation 3296) from a cross-sectional intellectual disabilities study [21] was used in the sample size calculation. The parent walking programme used to develop Walk Well had an approximate effect size of 3000 steps per day in a trial for adults who did not have intellectual disabilities [22]. To take account of the different population for this study, a target increase of 2500 steps/day and a standard deviation in the step count after the 12-week programme of 3500 were used for the sample size calculation. For 80 % power at the 5 % significance level, 32 participants per group were required. To allow for a dropout rate of 20 %, 40 participants in each group were required. No data were available to inform the likely degree of clustering. An increase in the study sample size of 20 % was decided upon, based on a conservative intraclass correlation coefficient of 0.1, and an average of 3 participants per cluster. Therefore, adopting a cautious approach, the final target sample size was 50 participants in each arm of the study.

## Intervention

### Walk well

Walk Well is an individual behaviour change intervention designed to support participants to make sustained changes in walking, increase overall physical activity levels and reduce sedentary behaviours. The overall aim of the programme was for participants to gradually increase their daily walking time by 30 min (equivalent to around 3000 steps) on at least five days of the week, by week 12.

The starting point for development of Walk Well was a parent walking programme shown to be effective in studies involving adults [22], adults older than 65 years [23] and as part of a multi-component weight loss programme for men at high risk of cardiovascular disease [24, 25]. The parent 12-week walking intervention was based on the transtheoretical and socio-cognitive models of behaviour change and included two individual physical activity consultations and a 12-week structured walking programme. The first physical activity consultation was focused on increasing motivation and reducing barriers to increase walking, with additional discussion of self-efficacy, decisional balance and techniques to support behaviour change [22]. Goal setting was used to agree a 12-week individualised graduated walking programme, in the form of a specially designed booklet and pedometer. A second physical activity consultation at the end of the 12-week intervention period focused on relapse prevention, encouragement and strategies to support behaviour change. No adults with intellectual disabilities took part in the trials of the parent walking programme.

The research team is experienced at adapting interventions to make them accessible to adults with intellectual disabilities and clinical groups. Our aims were to adapt the parent walking programme to take account of the cognitive and communication levels of adults with intellectual disabilities and to involve family and paid carers to support participants to make use of the programme. The parent walking intervention as simplified as much as possible by reducing the number of behaviour change techniques used. Small groups of adults with learning disabilities and carers were consulted about draft resources appropriate to the developmental level of adults with intellectual disabilities. Feedback from the groups was used to produce the final resources that were used in the Walk Well programme.

Walk Well involved three face-to-face meetings over a 12 week period between participants, carers where appropriate, and a walking advisor. Prior to the start of the intervention the walking advisor received training on communicating with adults with intellectual disabilities, motivational interviewing and delivering physical activity consultations.

The Walk Well physical activity consultation [26], based on the transtheoretical model and socio-cognitive models [27] of behaviour change, formed the basis of the meetings. The physical activity consultation had a semi-structured format and a person-centred approach [28] to ensure it was individualised to the needs of the participant with intellectual disabilities. We recognised the importance of reducing the complexity of the physical activity consultation and designed a behaviour change model with four core behaviour change techniques that are known to be effective in supporting individuals to increase levels of physical activity—mobilising social support for change, developing self-efficacy, goal setting and self-monitoring [20]. Although the emphasis was on these four components, the walking advisor tailored the physical activity consultation by drawing on additional behaviour change techniques such as identifying and overcoming barriers to change, and used a motivational interviewing approach where relevant.

Although it was recognized that carer involvement in the programme could be an important source of social support, participants had the final decision on whether to involve carers in the physical activity consultations. At the first meeting, the walking advisor gave accessible Walk Well educational booklets to participants and a separate booklet to carers. These resources were used to introduce the Walk Well programme and facilitate a discussion about potential benefits of walking. Following a discussion on the role of carers, and others, in providing social support and a check on participant self-efficacy, the walking advisor and participants developed an individualised six-week programme to progressively increase baseline step-counts, week on week. Participants were provided with an Omron Walking Style III pedometer (Omron Healthcare Inc, Illinois, U.S.A.) at the first meeting. The walking advisor provided training on how to use the pedometer and the Walk Well diary to self-monitor daily step counts against the agreed, individualised goals.

At the second meeting, participants reviewed their progress towards achieving the goals agreed at the first meeting by discussing the use of the pedometer and information recorded on the walking diary with the walking advisor. The walking advisor continued to use the physical activity consultation components to encourage behaviour change and to reinforce knowledge about the potential benefits of physical activity. Participants were invited to set progressive goals which were incorporated into a new six week, structured walking programme. The final meeting at 12 weeks focused on encouraging participants to maintain changes by reviewing goal attainment, perceived benefits discussing relapse prevention strategies to maintain increases in walking. Participants were given a certificate at the final meeting to show they had completed the Walk Well programme.

## Study outcomes

A researcher blinded to allocation collected all data at baseline, 12 weeks and 24 weeks. Demographic and self-reported health characteristics were collected using a structured interview schedule. Baseline postcode of residence was used to allocate participants to a category of socioeconomic status according to quintiles of Scottish Index of Multiple Deprivation (http://www.scotland.gov.uk/Topics/Statistics/SIMD).

### Primary outcome

The primary outcome was change in average number of steps walked per day at 12 weeks, measured using Actigraph GT3X accelerometers (manufacturing technology inc., Florida). Accelerometers are viewed as the gold standard method to measure physical activity and are reliable for the measurement of free-living step counts [29]. Adults with intellectual disabilities experienced minimal difficulties with the use of accelerometers in a previous study [30].

Participants were asked to wear the accelerometer during all waking hours for seven days, except when showering, bathing or swimming. To monitor wear time, participants and carers were asked to record the time when the accelerometer was put on each day, any periods when it was removed, and the time it was removed prior to going to bed. The minimum data requirement for inclusion in the analysis was six hours of data on at least three days from seven.

### Secondary outcomes

We used accelerometer data to measure total physical activity, moderate-vigorous physical activity and sedentary behaviours. The Walk Well protocol [20] used cut-offs from a previous intellectual disabilities weight loss study [30] to categorise accelerometer data as sedentary behaviour (0–499 counts per minute) and moderate-vigorous intensity activity (>1952 counts per minute). However, results for sedentary behaviour were calculated using a more recent consensus-based cut-off of less than 100 counts per minute [31]. Time spent in physical activity of any intensity was used as a measure of total physical activity (≥100 counts per minute).

Self-reported physical activity levels were collected using the International Physical Activity Questionnaire-Short version, a widely used measure of physical activity in the past seven days [32]. To take account of the study population, the researcher read the IPAQ-S questions to participants with support from carers where needed. The International Physical Activity Questionnaire-Short scoring protocol (available at https://sites.google.com/site/theipaq/scoring-protocol) was used to generate walking time (minutes/week), sitting time (minutes/week), and time spent in moderate and vigorous physical activity (minutes/week). To provide a global measure of overall activity levels the International Physical Activity Questionnaire-Short variables were combined to calculate total metabolic equivalent minutes/week [33].

Participants were invited to have their weight, height and waist circumference measured wearing light clothes without shoes. All measurements were made in duplicate and the final value calculated as the mean of the two measurements. Weight in kilograms (kg), was measured to the nearest 100 g (g), using SECA 877 scales (SE approval class III; SEA Germany). Height in metres (m) was measured to the nearest 1 mm (mm) using the SECA Leicester stadiometer (SECA, Germany). The height (m) and weight (kg) were used to calculate BMI using the formula; BMI = weight/height ^2^ (kg/m^2^). Waist circumference was measured to the nearest 0.5 cm (cm) at the mid-point between the iliac crest and the lowest rib, in full expiration with the participant standing.

The European Quality of Life-5 dimensions has been used in trials of walking interventions [22]. Most participants were unable to complete the European Quality of Life-5 dimensions visual analogue scale so only the health utility score from the five questions is reported. The nine item Subjective Vitality Scale [34], simplified for use by adults with intellectual disabilities assessed any positive effects of Walk Well on well being. To measure changes in self-efficacy the Self-Efficacy for Activity for Persons with Intellectual Disability [35] was completed at all three time points.

### Safety and adverse events

We assessed safety by reports of adverse events from participants and carers at meetings with the walking advisor and by the researcher asking participants about adverse events at each data collection point. Serious adverse events were classified as events that caused death, were life threatening, or necessitated admission to hospital.

## Statistical analysis

All statistical analysis programs in SAS (version 9.3) were developed prior to unblinding of the randomised groups, according to a Statistical Analysis Plan produced by the Glasgow Clinical Trials Unit. An intention to treat approach was used for the analyses, with all participants analysed in the groups to which they were randomised. The primary outcome, change in mean steps per day at 12 weeks from baseline, was analysed at the level of the individual, using mixed effects regression models taking account of clustering and adjusting for randomised group, baseline step count and the cluster size as used to stratify the randomisation. Similar regression models were fitted for secondary outcomes. Data are presented as Intraclass correlation coefficient, adjusted mean differences (95 % confidence interval) and corresponding p-values. For the primary outcome within group changes and between group changes were also calculated from a repeated measures mixed effects model.

## Results

### Baseline characteristics

**Fig. 1:**
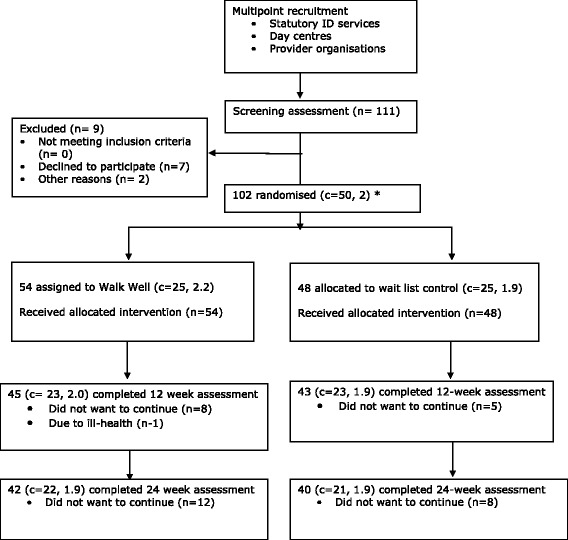
Screening, randomisation and completion of primary outcome measure. *number of clusters (mean size)

We randomised 102 participants in 50 clusters (Fig. 1); 54 to the Walk Well programme and 48 to the waiting list control group.

Demographic and health characteristics were similar in the two groups (Table 1), with slightly fewer people with mild intellectual disabilities in the waiting list control group. The rate of health problems reflects the complex needs of adults with intellectual disabilities [36]. A majority of participants lived in the most deprived neighbourhoods of Scotland. Both groups were in a sedentary category defined by mean counts less than 5000 steps per day [37] and 59 % of participants had a BMI in the obesity range (Table 2).

**Table 1:** Baseline characteristics of participants by randomised group. Values are numbers (percentages) unless otherwise stated

Variable	Walk Well (54)	Control (48)
	N (%)	N (%)
Female gender	25 (46.3 %)	20 (41.7 %)
Mean age (SD)	44.9 (13.5)	47.7 (12.3)
Intellectual disabilities		
Mild	37 (69.8 %)	21 (43.8 %)
Moderate	11 (20.8 %)	24 (50.0 %)
Severe	5 (9.43 %)	3 (6.3 %)
Type of support		
Lives independently	3 (5.5)	2 (4.2)
Family carer	29 (53.7)	23 (47.9)
Paid carer	22 (40.7)	23 (47.9)
SIMD quintile		
0–20 % most deprived	36 (66.7)	32 (66.7)
20–40 %	8 (14.8)	9 (18.8)
40–60 %	5 (9.3)	3 (6.3)
60–80 %	2 (3.7)	1 (1.9)
80–100 % least deprived	3 (5.6)	3 (6.3)
Diagnosis of epilepsy	3 (5.6 %)	7 (14.6 %)
Visual impairment	24 (44.4 %)	32 (66.7 %)
Hearing impairment	11 (20.4 %)	9 (18.8 %)
Mental ill-health	15 (27.8 %)	18 (37.5 %)
Problem behaviours	9 (20.0 %)	9 (19.2 %)
Weight status (BMI kg/m^2^)		
Normal weight (18.5–24.9)	7 (13.5 %)	10 (20.8 %)
Overweight (25–29.9)	16 (30.8 %)	8 (16.7 %)
Obesity (30–39.9)	22 (42.3 %)	22 (45.8 %)
Morbid obesity (>40.0)	7 (13.5 %)	8 (16.7 %)

*SD* standard deviation, *BMI* body mass index, *SIMD* Scottish Index of Multiple Deprivation

**Table 2:** Baseline primary and secondary outcomes of participants, by randomised group

	Walk Well		Control	
Outcomes	N	Mean (SD)	N	Mean (SD)
Primary outcome				
Step count per day	54	4744 (2076)	48	4818 (2784)
Secondary outcomes				
Percentage time per day PA	54	35.8 (10.4)	48	33.1 (11.3)
Percentage time per day MVPA	54	3.2 (2.7)	48	3.3 (2.9)
Percentage time per day sedentary	54	64.2 (10.5)	48	66.9 (11.3)
Total MET minutes/ week	53	1367.6 (1629.9)	40	1150.1 (1059.9)
Body mass index	52	32.3 (7.3)	48	32.6 (7.4)
Waist circumference	54	105.4 (16.5)	48	106.4 (18.3)
Subjective vitality	51	14.4 (2.7)	44	13.8 (2.9)
Self-efficacy	53	14.3 (3.1)	47	14.1 (3.0)
EQ-5D	53	0.8 (0.25)	48	0.7 (0.29)

*SD* standard deviation, *SE-AID* Self Efficacy and Intellectual Disabilities, *EQ-5D* European Quality of Life 5 Dimensions, *MET* metabolic equivalents, *PA* physical activity of any intensity, *MVPA* moderate vigorous physical activity

### Loss to follow up

In total, valid accelerometer data was available for 82 (80.4 %) of the 102 participants at the 12 week data collection point (Fig. 1). The proportion of participants lost to follow up was similar for the Walk Well (22.2 %) and control group (18.8 %) and there were no differences in baseline characteristics between participants lost to follow up and completers.

### Primary outcome

There was no significant post-intervention effect of Walk Well on mean steps per day at 12-weeks (adjusted group difference 69.5 steps per day 95 % confidence interval −1054 to 1193.3, *p =* 0.90; Table 3). The intraclass correlation for primary outcome was 0.50. No within group, pre-post intervention changes in steps per day were found for the intervention (adjusted difference 74.5 steps per day, 95 % confidence interval −551.1 to 700.20; *p =* 0.81) or control groups who participated in Walk Well at the end of the 12-week control period (adjusted difference −221.0 steps per day, 95 % confidence interval −915.7 to 473.62; *p =* 0.53).

**Table 3:** Main intention to treat analyses of effect of Walk Well programme on primary and secondary outcomes assessed immediately after end of programme (12 weeks)

	Walk Well		Control		Main between group comparison		
Outcomes	N	Mean (SD)	N	Mean (SD)	Intervention effect (95 % CI)^a^	p	ICC
Primary outcome							
Step count per day	42	4823 (2059)	40	4784 (2613)	69.5 (−1054, 1193.3)	0.90	0.51
Secondary outcomes							
Percentage time per day PA	42	33.5 (10.0)	40	34.0 (12.0)	- 1.5 (−6.1, 3.0)	0.5	0.22
Percentage time per day MVPA	42	3.0 (2.6)	40	3.1 (2.1)	0.3 (–0.7, 1.3)	0.55	0.42
Percentage time per day sedentary	42	66.4 (10.0)	40	65.9 (12.0)	1.6 (−3.0, 6.1)	0.49	0.22
Total MET minutes per week	37	1311.9 (1293.2)	37	1154.8 (1103.7)	56.0 (−428.8,540.9)	0.82	0.02
Body mass index	43	32.1 (7.7)	43	32.9 (7.5)	−0.21 (−0.83,0.41)	0.49	0.00
Waist circumference	45	104.9 (16.9)	42	107.8 (17.8)	−1.64 (−3.93,0.64)	0.15	0.00
Subjective vitality	39	14.6 (2.5)	35	14.3 (2.8)	0.33 (−0.85, 1.52)	0.57	0.00
Self-efficacy	43	14.4 (3.0)	42	13.7 (3.7)	0.77 (−0.68, 2.22)	0.29	0.08
EQ-5D	44	0.8 (0.27)	43	0.7 (0.30)	0.02 (−0.09, 0.14)	0.70	0.00

*SD* standard deviation, *ICC* Intraclass correlation coefficient, *SE-AID* Self Efficacy and Intellectual Disabilities, *EQ-5D* European Quality of Life 5 Dimensions, *MET* metabolic equivalents, *PA* physical activity of any intensity, *MVPA* moderate vigorous physical activity

^a^Between group mixed effects model adjusted for cluster and baseline value

Between 12 (post-intervention) and 24 weeks (follow-up), there was no within group change in the intervention group step count (adjusted difference 113.8 steps per day, 95 % confidence interval −552.3 to 779.75; *p =* 0.74).

### Secondary outcomes

There were no significant differences in any of the secondary outcomes attributable to participation in the Walk Well programme (Table 3).

### Adverse events

There were no adverse events associated with the trial.

### Compliance with the intervention

Seventy one percent of participants took part in all three planned face-to-face physical activity consultations with the walking advisor, 26 % took part in two and 3 % of participants one of the consultations.

## Discussion

Baseline characteristics of participants showed that their health is at risk because of low levels of physical activity, high levels of sedentary time and obesity. This trial found that the Walk Well programme had no effect on the mean steps/ day (primary outcome) or any of the secondary outcomes. Findings from qualitative interviews to gather participants’ views about the Walk Well programme and trial and a process evaluation will be published separately but showed that participants felt positive about taking part in the trial.

### Comparison to other studies

This is the first trial of a walking programme for adults with intellectual disabilities so we compared our findings to the two controlled trials of non gym-based programmes that aimed to increase physical activity levels [38, 39].

The Steps to Your Health (STYH) programme used a health education approach to increase moderate-vigorous physical activity of adults with intellectual disabilities using services offered by community based disability agencies, in three south-eastern states of the United States of America [38]. STYH included eight, weekly group sessions lasting 90 min, with each session focussed on a different health behavior topic. Partcipants were randomised to STYH (*n =* 216) or a hygiene and safety attention control group (*n =* 216). There was no effect of the STYH on moderate-vigorous physical activity or BMI. A programme in Stockholm County, Sweden [39] aimed to change the physical activity and diet of adults with mild-moderate intellectual disabilities living in group homes. The intervention comprised a ten session health education programme of participants, a ten session health behaviour study circle to increase knowledge and skills of paid carers and appointment of a health ambassador from the staff in each house. There was a statistically significant increase of 1608 steps/ day (*p =* 0.045) which may be partly attributable to the Stockholm programme having a greater number of face-to-face sessions with participants than Walk Well and a greater focus on changing the knowledge and behaviours of the paid carers.

### Strengths and limitations

The controlled design in this study minimised bias evident in the majority of non-gym based physical activity programmes [17]. A recruitment strategy (20) developed prior to the start of the trial overcame previously reported difficulties with the recruitment of participants with intellectual disabilities to randomised controlled studies [40]. This resulted in a large, representative sample with similar health characteristics [36] and deprivation levels [41] to a population-based sample from the same geographical area. With primary outcome data for 80 % of participants the results reported here are less likely to be biased than the findings in the Steps to Your Health and Stockholm trials which had primary outcome data available for only 26.6 % [38] and 49.6 % of participants [39], respectively.

Participants in Walk Well found the subjective questionnaires difficult to complete. Using accelerometers to collect objective physical activity data solves the problem of gathering reliable self-report data using the International Physical Activity Questionnaire-Short. A version of the European Quality of Life-5 Dimensions has been developed for use by adults with intellectual disabilities but still uses the visual analogue scale that most participants could not complete. Even the Self-Efficacy for Activity for Persons with Intellectual Disability [35] developed specifically for use by adults with intellectual disabilities was too complex for many participants.

### Possible explanations for study findings

Individual, social and environmental factors [42] can help to explain why the Walk Well programme was not effective.

The lack of effect is partially explained by the challenges in adapting complex behaviour change interventions for adults with intellectual disabilities. In keeping with guidelines for developing physical activity interventions for disadvantaged groups [43] we developed accessible resources and simplified the programme as much as possible. The the Steps to Your Health [38] and Stockholm [39] programmes used a straightforward health education approach and did not include the more complex behaviour change techniques included in Walk Well, such as self monitoring or goal setting. Many participants and carers expressed difficulties using the pedometers and walking diary to self monitor daily step count against their individual goals. Therefore, it could be that the behaviour change techniques [44] that contributed to the effectiveness of the parent walking programme [22–25] are too complex and abstract for most adults with intellectual disabilities.

The additional barriers to physical activity experienced by adults with intellectual disabilities mean that decision making and actions are most often expressed in the context of existing personal relationships and the majority of participants in Walk Well were supported by family or paid carers during walking. However, many participants and carers reported difficulties finding time to walk together. Cuts in social care budgets have disproportionately impacted on disabled people [45] and even when social care support is available, it is often not funded at a level that allows paid carers to support adults with intellectual disabilities to be physically active [46–48]. Therefore, the lack of effect in the Walk Well trial may be due to a lack of availability of social support [49] to make walking accessible [46], facilitate community participation [50] and moderate social disadvantages [51]. Additional support from external organisations [51] could reduce the social capital/ networks barriers to adults with intellectual disabilities participating in community activities caused by reductions in social care support [52]. For example, social enterprises, volunteer organisations and buddy programmes may all have a role to play in supporting adults with intellectual disabilities to be more active.

The Walk Well trial successfully recruited a sample representative of the multiple social disadvantages experienced by adults with intellectual disabilities. This meant that a far higher proportion of participants in Walk Well lived in deprived neighbourhoods compared to the trials of the parent walking intervention [22–25]. Based on the PROGRESS-Plus [53] framework for health equity (place of residence, race/ethnicity, occupation, gender, religion/culture, education, socio-economic status, social capital/networks, disability, sexual orientation, and age) adults with intellectual disabilities are often multiply disadvantaged by disability, place of residence, socio-economic status [7] and social capital/ networks [49]. Older adults are the only PROGRESS-Plus group that walking programmes have been shown to be effective for but this evidence is based on samples living in the least deprived neighbourhoods. For example, 2.4 % of the sample in the trial of the parent walking programme in older adults [23] lived in the most deprived quintile and less than 10 % of older people in the PACE-Lift trial in primary care [54]. Since it appears that trials of walking programmes have recruited samples at relative social advantage, one interpretation of our findings could be that walking programme effectiveness is not generalisable to socially valid populations or groups who experience multiple social disadvantage.

### Unanswered questions and future research

Finding ways to support adults with intellectual disabilities to increase their levels of physical activity and reduce time spent sedentary is still a health improvement priority.

There is a recognised need to derive valid theoretical models of behaviour change as a necessary pre-cursor to designing any effective physical activity programmes [46]. Participants in Walk Well experienced difficulties that with self-monitoring and goal setting. Therefore, part of the process to develop theoretical models should examine whether behaviour change techniques can be adapted to make them accessible for adults with intellectual disabilities.

The number of face-to-face sessions in Walk Well was greater than in the parent walking programme. However, most walking programmes that target individuals who are as sedentary as the Walk Well sample have three or more sessions a week for the duration of the intervention sessions [19]. Both previous controlled trials of non gym-based physical activity programmes also included more intensive interventions, with eight-ten health education group sessions for adults with intellectual disabilities, supplemented with weekly walking groups [38] or sessions for carers [39]. Therefore, research should examine the effectiveness of more intensive walking programmes than Walk Well and have a greater focus on changing carer knowledge and behaviour [39].

Participants in Walk Well had daily step counts in the sedentary range and were had accelerometer counts in the sedentary range for 65 % of the time. Given the social capital/ network barrier to walking experienced by participants in the Walk Well trial, an alternative approach to improving the health of adults with intellectual disabilities is to design programs to reduce time spent sitting [55]. The replacement of sitting time with standing or light intensity physical activity is associated with health improvement and reduced cardiometabolic risk [56]. However, the acceptability and feasibility of programmes to reduce sitting time for adults with intellectual disabilities needs to be examined.

The broader challenge arising from the Walk Well trial is how to develop walking programmes that address the impact on health of multiple social disadvantage [57]. Since there is minimal evidence that walking programmes are effective for socially disadvantaged groups, if walking programmes are implemented in health and other services based on existing evidence they could increase the health inequalities of disadvantaged groups [4, 58]. Therefore, the Walk Well trial highlights the need for research to examine the feasibility and effectiveness of walking programmes for disadvantaged groups within communities.

## Conclusions

This study demonstrated that it is feasible to recruit and retain a large sample of adults with intellectual disabilities who would benefit from becoming more active and less sedentary. However, the Walk Well programme for adults with intellectual disabilities did not change walking or any of the secondary outcomes. Therefore, it should not be assumed that physical activity interventions with proven efficacy can be easily adapted for adults with multiple and complex patterns of social disadvantage. Social support from others should be conceptualised as a central component of physical activity programmes to support adults with intellectual disabilities. However, increased participation in walking and other types of physical activity is likely to require specific social support that is over and above existing support from family and paid carers.

## Additional file

Additional file 1:
**CONSORT 2010 checklist of information to include when reporting a cluster randomised trial.** (DOCX 25 kb)
